# Correction: Preparation of UV-curable PSAs by grafting isocyanate-terminated photoreactive monomers and the effect of the functionality of grafted monomers on the debonding properties on Si wafers

**DOI:** 10.1039/d3ra90045b

**Published:** 2023-05-16

**Authors:** Hee-Woong Park, Hyun-Su Seo, Kiok Kwon, Seunghan Shin

**Affiliations:** a Green Chemistry & Materials Group, Korea Institute of Industrial Technology (KITECH) Cheonan Chungnam 31056 Republic of Korea shshin@kitech.re.kr; b Department of Green Process and System Engineering, University of Science & Technology (UST) Daejeon 34113 Republic of Korea

## Abstract

Correction for ‘Preparation of UV-curable PSAs by grafting isocyanate-terminated photoreactive monomers and the effect of the functionality of grafted monomers on the debonding properties on Si wafers’ by Hee-Woong Park *et al.*, *RSC Adv.*, 2023, **13**, 11874–11882, https://doi.org/10.1039/D3RA00398A.

The authors regret that an incorrect grant number was shown in the acknowledgements section of the published article. The corrected section should read:

This study was supported by the Technology Development Program (S2830047) by the Ministry of SMEs and Startups (MSS) and by Technology Development Project for Safety Management of Household Chemical Products (2022002980010) by the Ministry of Environment (ME), Republic of Korea.

The Royal Society of Chemistry apologises for these errors and any consequent inconvenience to authors and readers.

## Supplementary Material

